# Predictors of PrEP retention in at risk patients seen at a HIV primary care clinic in San Diego

**DOI:** 10.1177/09564624231179276

**Published:** 2023-06-04

**Authors:** Shayna Herns, Rebecca Panwala, Allan Pfeil, Manuel Sardinha, Vito Rossi, Jill Blumenthal, Lucas Hill

**Affiliations:** 1 8784 University of California San Diego, San Diego, CA, USA; 2 12262 University of Massachusetts, Chan Medical School, Worcester, MA, USA; 3 6556 Philadelphia College of Osteopathic Medicine, Philadelphia, PA, USA

**Keywords:** HIV (human immunodeficiency virus), retention, prevention, viral disease

## Abstract

**Background:**

Adherence to medication and retention in care are key contributors to the efficacy of pre-exposure prophylaxis (PrEP) for prevention of HIV. Therefore, it is important to understand factors that may impact retention in various settings that prescribe PrEP.

**Methods:**

We evaluated factors associated with retention in care 3 and 12 months after PrEP initiation at a primary care HIV clinic in San Diego. Retention was defined as having an office/virtual visit within 1 month from the 3- or 12-months time point or interacting with the clinic leading to medication being refilled.

**Results:**

A total of 199 patients were included. Retention rates were 74.4% and 52.8% at 3 and 12 months respectively. In the multivariate analysis, reporting depression or anxiety was associated with being retained in care (*p* = 0.004) and identifying as cisgender female was associated with lack of retention (*p* = 0.04) at 3 months. Testing positive for a sexually transmitted infection was associated with 12-months retention (*p* = 0.004); however, this was likely influenced by difference in the frequency of testing in those retained versus not retained.

**Conclusion:**

Ongoing efforts to determine the optimal method for provision of PrEP care that supports retention for different populations at risk for HIV, are needed.

## Introduction

Once daily pre-exposure prophylaxis (PrEP) for the prevention of HIV acquisition has been shown in randomized controlled trials and open label studies to be highly efficacious in preventing HIV acquisition if taken as prescribed.^1–3^ However, results from several studies have highlighted the importance of adherence to daily pill taking. Findings from these studies have demonstrated reduced PrEP efficacy in the setting of low adherence to PrEP.^4,5^ Various studies have examined facilitators of and barriers to PrEP adherence, including patient demographics, mental health conditions, and sociobehavioral factors.^6–8^ In addition to medication adherence, retention in PrEP care, including the need to complete required laboratory follow-up and obtain prescription refills, is essential for effective and safe provision of PrEP. Efforts to continue to evaluate and understand factors associated with medication adherence and retention in PrEP care are needed.

More recently, multiple clinical sites in the United States have begun to examine PrEP adherence and retention in real-world settings as opposed to clinical trials, seeking to identify potential areas of intervention to improve engagement in the continuum of PrEP care.^9–14^ In these studies, a recurring obstacle to PrEP adherence and retention was structural factors, namely cost, insurance coverage and/or access to PrEP financial assistance programs.^9–11^ Patient demographics have also been examined, but they have yet to demonstrate a significant difference in retention by race/ethnicity or age.^10,12^ However, studies have shown that in patients initiating PrEP, young age, Black race or Hispanic ethnicity, and smoking were associated with less than 60% PrEP adherence, and that assigned female sex at birth was associated with complete PrEP discontinuation.^13,14^ While social factors, including stimulant drug and alcohol use, have been shown to increase HIV acquisition due to association with high risk sexual risk behaviors, they have not reliably been shown to reduce PrEP adherence or retention.^11,14,15^ Comorbid conditions, including depression, anxiety, diabetes, hypertension, asthma, and others have been associated with increased retention, but concurrent diagnoses of STIs have shown mixed results, with some studies demonstrating significantly reduced retention and others showing no significant difference.^10,12^ While specific findings of factors associated with poor PrEP adherence and/or retention have varied, nearly universally these studies demonstrate poor overall retention.

Prior studies have been conducted at a variety of clinic types, including primary care offices, community-based programs, STI, lesbian/gay/bisexual/transgender/queer (LGBTQ+) and infectious disease specialty clinics.^9–11^ However, there are limited data examining PrEP adherence and retention in specialty HIV care clinics. The purpose of this study was to contribute to growing literature on the PrEP care continuum and identify factors associated with PrEP retention. This retrospective study analyzed data from a cohort of patients seen at UC San Diego Health (UCSD)’s Owen Clinic, a comprehensive primary care center for HIV care and prevention, including initiation and follow-up of PrEP.

## Materials and Methods

This was a retrospective cohort study in which data were collected from patients who initiated PrEP between January 2018 and December 2020 at the UCSD Owen Clinic. Exclusion criteria included patients who initiated PrEP prior to 2018 and patients referred to the Owen Clinic for post exposure prophylaxis (PEP) therapy. Retention was defined as having an office/virtual visit up to 1 month before or 1 month after the defined time point (3, 6, or 12 months after initiating PrEP) or interacting with the clinic leading to medication being refilled. Interactions with the clinic could have included electronic messaging or phone calls to the clinic. Short- and long-term retention were defined as retention as above at months 3 and 12, respectively. Although outcomes focused on 3- and 12-months retention, 6-months retention data were collected as well to enhance the ability to describe the trend over time. Data were collected via electronic health record (EHR) using a standardized case report form. Patient characteristics including sociodemographics and risk behaviors were collected from an initial visit and at months 3, 6 and 12 from the start of PrEP therapy. Self-reported adherence was also measured at these time points and obtained via provider progress notes which documented missing ≤3 PrEP doses per month, missing >3 PrEP doses per month, or completely stopping PrEP. Sociodemographics and risk behaviors of those that were and were not retained were compared using Mann-Whitney U or chi-square tests including chi-square contingency tables for variables with multiple entries. Stepwise multivariate logistic regression was performed to identify predictors of retention, which included age, race, ethnicity, either sex assigned at birth or gender identity, depression/anxiety, sexual history, primary insurance, and number of partners in the last 3 months. Engaging in methamphetamine use, alcohol use, and STI testing results were excluded from the short-term retention model due to lack of available data; however, STI testing results were included in the model for long term retention. Statistical analysis was completed using MedCalc Version 19.1.3. This study was approved by the Institutional Review Board at the University of California San Diego, Project#210417.

## Results

One-hundred and ninety-nine patients were included in the analysis, with a median age of 32 years old (IQR 27–39). Eighty eight percent of patients identified as male and 94% were assigned male at birth, with 36.2% of patients identifying as Hispanic and 81.9% reporting sex with men only. Other baseline patient demographics are shown in Table 1. Overall retention rates were 74.4% at 3 months (*n* = 148), 62.8% at 6 months (*n* = 125), and 52.8% at 12 months (*n* = 105). When comparing retention rates by year of PrEP initiation there was no significant difference in 3- and 6-months retention rates for those starting in 2018, 2019, or 2020. However, 12-months retention rates did significantly differ by year of initiating treatment, with 58.1%, 47.3%, and 60.3% being retained in those starting PrEP in 2018, 2019, and 2020 respectively (*p* = 0.006).

**Table 1.** Predictors of short-term retention (*n* = 199).

Characteristics	Retained (*n* = 148)	Not retained (*n* = 51)	Total (*n* = 199)	*p*-value (univariate)
Median age (IQR)	32 (27–39)	30 (25–38)	32 (27–39)	0.24
Race (%)				
White	80 (54.1)	30 (58.8)	110 (55.3)	0.91
Black	2 (1.4)	1 (2.0)	3 (1.5)	
API/AI	16 (10.8)	4 (7.8)	20 (10.1)	
Other/did not disclose	46 (31.1)	16 (31.4)	62 (31.2)	
Not available	4 (2.7)	0 (0.0)	4 (2.0)	
Ethnicity (%)				
Hispanic	52 (35.1)	20 (39.1)	72 (36.2)	0.62
Non-Hispanic	89 (60.1)	29 (56.9)	118 (59.3)	
Not reported	7 (4.7)	2 (3.9)	9 (4.5)	
Sex assigned at birth (%)				
Male	141 (95.3)	46 (90.2)	187 (94.0)	0.19
Female	7 (4.7)	5 (9.8)	12 (6.0)	
Gender identity (%)				
Cisgender man	131 (88.5)	43 (84.3)	174 (87.4)	0.48
Cisgender woman	6 (4.1)	5 (9.8)	11 (5.5)	
Transgender/Non-binary	7 (4.7)	2 (3.9)	9 (4.5)	
Other/prefer not to disclose	4 (2.7)	1 (2.0)	5 (2.5)	
Baseline depression or anxiety (%)				
Yes	40 (27.0)	4 (7.8)	44 (22.1)	0.004
No	108 (73.0)	47 (92.2)	155 (77.9)	
Active/history of methamphetamine use (%)				
Yes	8 (5.4)	2 (3.9)	10 (5.0)	0.72
No	90 (60.8)	30 (58.8)	120 (60.3)	
Not reported	50 (33.8)	19 (37.7)	69 (34.7)	
Current alcohol use (%)				
Yes	88 (59.5)	30 (58.8)	118 (59.3)	0.65
No	19 (12.8)	8 (15.7)	27 (13.6)	
Not reported	41 (27.7)	13 (25.5)	54 (27.1)	
Sexual history (%)				
Sex with women only	2 (1.4)	2 (3.9)	4 (2.0)	0.67
Sex with men only	121 (81.8)	42 (82.4)	163 (81.9)	
Sex with women and men	19 (12.8)	5 (9.8)	24 (12.1)	
Other/not available	6 (4.1)	2 (3.9)	8 (4.0)	
Primary insurance (%)				
Medicaid	30 (20.3)	14 (27.5)	44 (22.1)	0.53
Commercial	61 (41.2)	21 (41.2)	82 (41.2)	
PrEP assistance program	53 (35.8)	14 (27.5)	67 (33.7)	
Medicare	3 (2.0)	2 (3.9)	5 (2.5)	
None	1 (0.7)	0 (0.0)	1 (0.5)	
Any positive STI result (%)				
Positive	14 (9.5)	4 (7.8)	18 (9.04)	0.81
Negative	88 (60.3)	29 (56.9)	117 (58.8)	
Testing not performed	46 (31.2)	18 (35.3)	64 (32.2)	
Median number of sexual partners in prior 3 months (IQR)	3 (1–6)	2 (1–5)	3 (1–6)	0.14

In the univariate analysis, self-report of depression or anxiety was significantly associated with short-term retention (*p* = 0.004). In the multivariate analysis having depression or anxiety remained associated with short-term retention [*p* = 0.004, odds ratio (OR) 5.31 (1.69–16.67)], while identifying as cisgender female was associated with lack of retention [*p* = 0.04, OR 0.24 (0.06–0.95)]. In the univariate analysis individuals reporting methamphetamine use (*p* = 0.03) and individuals assigned female at birth were less likely to be retained long-term (*p* = 0.047), whereas individuals with a positive STI test were more likely to be retained long-term (*p* = 0.003) (Table 2). In the multivariate analysis having a positive STI result remained significant for being retained long-term [*p* = 0.004, OR 2.84 (1.41–5.72)]. However, when controlling for the frequency of STI testing and comparing the rate of positivity, there was no statistically significant difference between the two groups (0.2 vs 0.14, *p* = 0.2).

**Table 2.** Predictors of long-term retention (*n* = 199).

Characteristics	Retained (*n* = 105)	Not retained (*n* = 94)	*p*-value (univariate)
Median age (IQR)	32 (27–39)	30 (26–39)	0.91
Race (%)			
White	58 (55.2)	52 (55.3)	0.80
Black	2 (1.9)	1 (1.1)	
API/AI	9 (9.6)	11 (11.7)	
Other/did not disclose	31 (29.5)	24 (25.5)	
Not available	5 (4.8)	6 (6.4)	
Ethnicity (%)			
Hispanic	37 (35.2)	35 (37.2)	0.88
Non-Hispanic	62 (59.0)	56 (59.6)	
Not reported	6 (5.7)	3 (3.2)	
Sex assigned at birth (%)			
Male	102 (97.1)	85 (90.7)	0.047
Female	3 (2.9)	9 (9.6)	
Gender identity (%)			
Cisgender male	95 (90.5)	79 (84.0)	0.33
Cisgender female	3 (2.9)	8 (8.5)	
Transgender/Non-binary	4 (3.8)	5 (5.3)	
Other/prefer not to disclose	3 (2.9)	2 (2.1)	
Baseline depression or anxiety (%)			
Yes	24 (22.9)	20 (21.3)	0.79
No	81 (77.1)	74 (28.7)	
Active/history of methamphetamine use (%)			
Yes	2 (1.9)	8 (8.5)	0.03
No	67 (63.8)	53 (56.4)	
Not reported	36 (34.3)	33 (35.1)	
Current EtOH use (%)			
Yes	62 (59.0)	56 (59.6)	0.68
No	13 (12.4)	14 (14.9)	
Not reported	30 (28.6)	24 (25.5)	
Sexual history (%)			
Sex with women only	3 (2.9)	1 (1.1)	0.54
Sex with men only	86 (81.9)	77 (81.9)	
Sex with women and men	11 (10.5)	13 (13.8)	
Not available/other	5 (4.8)	3 (3.2)	
Primary insurance (%)			
Medicaid	19 (18.1)	25 (26.6)	0.15
Commercial	44 (41.9)	38 (40.4)	
PrEP assistance program	41 (39.0)	27 (28.7)	
Medicare	1 (1.0)	4 (4.3)	
Any positive STI result (%)			
Positive	41 (39.0)	14 (14.9)	0.003
Negative	63 (60.0)	61 (64.9)	
Testing not performed	1 (1.0)	19 (20.2)	
Median number of sexual partners in prior 3 months (IQR)	3 (1–6)	3 (1–5)	0.27

Self-reported adherence was assessed at each clinic visit. At the 3-months time point, 68% (101/148) of those retained reported missing ≤3 PrEP doses per month, 12% (18/148) reported missing >3 PrEP doses per month, and 20% (29/148) were either off PrEP or adherence was not reported. Self-reported adherence at the 12-months time point was similar, of which 64% (67/105) of those retained reported missing ≤3 PrEP doses per month, 10% (10/105) reported missing >3 PrEP doses per month, and 27% (28/105) were either off PrEP or adherence was not reported.

Patients were also evaluated for PrEP side effects at each visit. The number of self-reported side effects declined over time, with 16% (24/148) of patients reporting at least one side effect at 3 months, then 7% at both the 6-months (9/125) and 12-months (7/105) time points. The majority of patients did not report any side effects of PrEP, with 68% (100/148) denying any side effects at 3 months, 66% (82/125) at 6 months and 80% (84/105) at 12 months.

## Discussion

We sought to identify predictors of PrEP retention in patients seen in a comprehensive HIV clinic in San Diego, CA. In this cohort of patients, retention declined over time to about 50% at 1 year, similar to that of other real-world data.^10,11,12^

We found that identifying as cisgender female was associated with a lack of retention in care. Similar findings have been demonstrated in other studies in which cisgender women were found more likely than cisgender men to discontinue PrEP.^13^ An open-label demonstration project of PrEP use among US cisgender women also found that PrEP adherence and retention was lower in cisgender women relative to similar studies focusing on men who have sex with men (MSM) populations.^16^ Previous studies examining PrEP attitudes among women have identified negative stereotypes about individuals who use PrEP and stigma around using PrEP as barriers to uptake.^17,18^ Confronting these attitudes may require a sustained effort from providers throughout treatment, especially if such beliefs are shared among and reinforced by peers after PrEP has been initiated. Future studies may consider evaluating if women may be better retained in reproductive health clinics that integrate sexual health or by their primary care physician as opposed to HIV clinics. Having a diagnosis of depression or anxiety was found to be associated with short-term retention. A recent review article identified depression and anxiety as potential barriers to PrEP adherence with mixed results regarding impact on retention; however, they also found that taking PrEP is associated with reductions in anxiety, which may partially explain the results in our study.^19^ One previous study observed increased PrEP adherence among cisgender MSM with moderate depression/anxiety, although this effect was reversed among MSM with severe depression/anxiety, who demonstrated significantly less adherence. The authors speculate that moderate symptoms may contribute to concern about HIV infection, thereby facilitating adherence, whereas severe symptoms may cause a diminished sense of agency or capacity for self-care, creating a barrier to adherence. This hypothesis could be extended to explain improved retention among these patients, although the effects of depression and anxiety on long-term PrEP retention remain unclear.^20^ We hypothesize that PrEP care may also provide patients with underlying depression or anxiety an opportunity to see a medical provider to discuss symptoms and treatment, increasing their likelihood of attending PrEP visits.

Although having a positive STI result was associated with long-term retention, this finding was likely due to more frequent STI testing in those that were retained and thus more positive results. This was supported by controlling for frequency of testing between the two groups in which there was no difference in the frequency of positive STI results.

Reporting a history of or active methamphetamine use was associated with lack of long-term retention in the univariate analysis. While some studies have shown an association between drug/alcohol use and discontinuation of PrEP, this finding has not been reliably demonstrated in other studies.^13,14,15^ A recent study found that individuals with a substance use disorder were more likely to experience gaps in the PrEP continuum of care.^21^ However, our observation was limited by a large percentage of patients with missing data regarding methamphetamine use, making it difficult to draw any firm conclusions.

This study has several limitations, including a relatively small number of cisgender women and females assigned at birth included in the study given the general demographics of those at risk of HIV in San Diego. In addition, a few important limitations of this study pertain to the effects of the COVID-19 pandemic. Prior to the pandemic, patients presented for clinical visits and STI testing in-person at 3-, 6- and 12-months time points. Due to the pandemic, some patients opted to move in-person visits to telemedicine, which may have impacted their likelihood of presenting for in-person STI testing. Further, due to concerns regarding COVID-19, a number of patients reported decreasing sexual activity, with some abstaining from sex altogether. Due to these changes in sexual activity, some patients perceived risk of acquiring HIV changed, leading to some patients stopping PrEP. We evaluated retention rates by year of PrEP initiation to try to assess the impact of the COVID-19 pandemic on retention, with the lowest 12-months retention rates being in those that started PrEP in 2019. This may reflect the 12-months follow up occurring after the COVID-19 pandemic started leading to lower retention. However, 12-months retention was actually highest in those that started PrEP in 2020 which may have been influenced by the role out of telemedicine after he start of the pandemic. Finally, the study was retrospective, resulting in higher rates of missing data for variables such as methamphetamine and alcohol use as well as an inability to assess adherence and side effects to PrEP in those not retained.

## Conclusion

In examining predictors of PrEP retention at a comprehensive HIV care clinic, identifying as cisgender female was associated with a lack of retention in care. In addition, baseline anxiety/depression was significantly associated with short-term PrEP retention. It will be important for ongoing research to identify optimal methods for PrEP delivery, such as integrated reproductive health or primary care clinics, which may be better suited for PrEP retention in these populations.
